# Mandibular Advancement Devices in OSA Patients: Impact on Occlusal Dynamics and Tooth Alignment Modifications—A Pilot Prospective and Retrospective Study

**DOI:** 10.3390/dj12110370

**Published:** 2024-11-19

**Authors:** Aylin Uyaner, Helen Schneider, Aditya Parikh, Kathrin Paeske-Hinz, Anna Konermann

**Affiliations:** 1 Department of Orthodontics, University Hospital Bonn, 53111 Bonn, Germany; 2 Fraunhofer—Institute for Intelligent Analysis and Information Systems (IAIS), Sankt Augustin 53757, Germany; 3 Private Practice, Herne 44623, Germany

**Keywords:** mandibular advancement devices, obstructive sleep apnea, occlusal changes, overbite, overjet, side effects

## Abstract

**Background**: The widespread prevalence of obstructive sleep apnea (OSA) underscores the necessity for effective therapies. Mandibular advancement devices (MADs) have emerged as valid treatment for mild to moderate cases, despite the associated dental side effects. **Methods**: This study evaluates the nature, onset, and long-term manifestation of these side effects. In the prospective group (*n* = 12), dental impressions were taken pre-MAD-insertion and at intervals of three, six, nine, and twelve months post-insertion to monitor occlusal alterations. In the retrospective group, participants (*n* = 8) wearing MADs for 7 years at average underwent lateral cephalogram assessments to compare with pre-treatment X-rays. All participants completed a specific questionnaire. Statistical analysis was performed via *t*-test and with *p* < 0.05 as the significance level. **Results**: The majority of participants in both groups consistently used MADs and reported significant sleep quality improvements, rating common symptoms like jaw tension as negligible. In both the prospective group and the retrospective group, significant reductions in overjet were observed at multiple time points, with the prospective group showing reductions at six months (*p* = 0.001), nine months (*p* > 0.001), and twelve months (*p* = 0.019), while the retrospective group indicated a notable decrease between baseline and follow-up assessments after a mean of seven years of device wear (*p* = 0.004). A slight overbite increase of 0.2 mm was prospectively observed after one year, whereas a trend towards a minimal decrease over the long term was observed in the retrospective sample (*p* = 0.003). Noteworthy changes in angle class or lower incisor inclination were absent. Cephalograms revealed significant IOK-NL angle alterations with a mean of 98.2° before and 95.2° upon long-term treatment (*p* = 0.020). **Conclusions**: These findings suggest that MADs are effective in treating OSA with minor adverse effects. This study advocates for moderate mandibular protrusion to balance therapeutic efficacy with dental health considerations, crucial for optimizing treatment outcomes. Nonetheless, the limited sample size warrants caution when generalizing these results to the broader population.

## 1. Introduction

Sleep disorders and their implications for overall well-being have become increasingly pertinent in contemporary society and epidemiological research indicates that approximately one third of the global population experiences sleep disorders [1]. The American Academy of Sleep Medicine (AASM) updated and reissued the International Classification of Sleep Disorders (ICSD) in 2014, organizing it into six distinct categories comprising insomnia disorders, sleep-related breathing disorders (SRBDs), central disorders of hypersomnolence, circadian rhythm sleep–wake disorders, parasomnias, and sleep-related movement disorders. SRBDs encompass sleep apnea, which can be categorized into central and obstructive forms. In SRBDs, apnea episodes occur during sleep, defined as temporary pauses in breathing lasting several seconds and considered clinically significant when persisting for at least 10 s and reoccurring at least five times per sleep cycle [2]. Apnea can manifest as central or obstructive, where central sleep apnea (CSA) represents the less common variant, with a prevalence not exceeding 20% [3,4]. Unlike obstructive sleep apnea (OSA), as the second form, CSA does not involve upper airway obstruction but results from a disruption in the central respiratory drive while the airways remain unobstructed. OSA, classified into adult and childhood subtypes, exhibits gender-based prevalence discrepancies of 2% in women and 4% in men, with recent estimates suggesting there are approximately 936 million individuals aged 30 to 69 experiencing mild OSA and 425 million within the same age range enduring moderate to severe OSA worldwide [5,6]. The global prevalence of OSA is estimated at 54%, with a high body mass index (BMI), advancing age, and male gender identified as significant risk factors in the literature; however, these covariates do not mitigate the pre-existing heterogeneity observed in the population [7]. The prevalence of OSA is moreover notably higher in specific patient populations, including hypertensive individuals, those with coronary disease, and candidates for bariatric surgery. OSA adversely affects quality of life and is associated with various negative safety and long-term health outcomes, including cardiovascular disease and the development of diabetes. Additionally, short habitual sleep duration can lead to excessive daytime sleepiness [8]. OSA involves pharyngeal collapse due to upper airway obstruction [6]. During sleep, relaxation of the pharyngeal muscles narrows the airway, with disrupted muscle stimulation patterns leading to collapse, while relaxation of the genioglossus muscle further worsens obstruction [9,10]. Untreated OSA poses risks of chronic hypertension, cardiovascular events, and stroke alongside the impairments in daily life and relationships [11]. Polysomnography revealing an Apnea–Hypopnea Index (AHI) of 15 events per hour or more, coupled with symptoms such as snoring, excessive tiredness, poor concentration, or comorbidities like hypertension or atrial fibrillation warrants treatment [12,13,14]. The therapeutic options for OSA are broad and interdisciplinary, and categorize into invasive and non-invasive approaches dependent on OSA severity, patient history, and compliance. Non-invasive methods include treatment with functional appliances, such as the wearing of a continuous positive airway pressure (CPAP) mask and mandibular advancement devices (MADs), whereas invasive treatments encompass nasal surgery, pharyngoplasty, rapid maxillary expansion, bimaxillary repositioning osteotomy, and hypoglossal nerve stimulation [15,16,17,18,19,20]. CPAP therapy is suggested for moderate to severe OSA, offering significant benefits and being considered the gold standard; however, it frequently encounters poor tolerance, with just half of patients maintaining adherence beyond a year, largely attributed to issues such as device noise, dryness of the oral and upper respiratory tract, and facial pressure sores [21,22].

The second non-invasive treatment option represents MADs consisting of custom-made bimaxillary splints fabricated by dentists to advance the mandible during sleep, thereby effectively opening the upper airway [23]. MADs are bimaxillary anchored plastic splints, interconnected to maintain the lower jaw anteriorly, with initial impressions determining the jaw relation at approximately 50% protrusion distance, followed by securing the splints with subsequent protrusion elements for readjustment by the dentist. While CPAP therapy retains its status as the preferred method for managing OSA due to its efficacy in reducing AHI and enhancing blood oxygen saturation, MADs emerge as the treatment of preference for individuals with mild to moderate OSAS and in cases of CPAP intolerance, which is underscored by the superior patient compliance and increased usage of MADs, as they are better tolerated by patients [24,25]. However, MAD treatment was found to exhibit significant side effects, particularly impacting occlusion. A 10-year follow-up study on obstructive sleep apnea therapy revealed substantial dental side effects, suggesting potential progression over time, with MAD treatment showing more pronounced changes in dental occlusion compared to CPAP therapy [26]. The observed side effects included the retroclination of upper incisors, proclination of lower incisors, reduced overjet, and overbite, as well as alterations in the total occlusal contact area, potentially contributing to poor patient compliance and therapy dropouts [27].

Further research is essential to better understand the factors influencing the occurrence and severity of these side effects as well as their long-term implications on patient treatment outcomes.

Therefore, this combined prospective and retrospective study aimed to thoroughly assess the occurrence and severity of side effects to enhance the effectiveness and acceptance of MADs as a therapy for OSA. The prospective part of the investigation sought to pinpoint the timing of dental side effects, such as changes in dental alignment and bite position, through rigorous patient screening closely meshed every three months after MAD insertion. Meanwhile, the retrospective analysis aimed to evaluate the long-term impact on tooth position changes after at least two years of MAD treatment. By investigating these aspects, valuable insights can be gained to minimize dental complications while optimizing therapeutic strategies and outcomes, thereby improving the overall care for individuals with OSA.

## 2. Materials and Methods

This study was performed according to the ethical principles of the World Medical Association Declaration of Helsinki. Informed consent was obtained from all participants included in the study. This study has been independently reviewed and approved by the Ethical Committee of the University of Bonn, Germany (reference number 322/22).

### 2.1. Patient Groups

The prospective group consisted of study participants for whom the use of a MAD was indicated for OSA treatment. The inclusion criteria encompassed consistent MAD usage and attendance at a minimum of three of the scheduled screening appointments every three, six, nine, and twelve months. The exclusion criteria included a lack of interest in the study, transition to continuous positive airway pressure (CPAP) therapy and voluntary treatment discontinuation. Among the initial cohort of 28 patients included in the study, 12 met the defined criteria for subsequent analysis. The study cohort comprised four female and eight male participants with an average age of 59.7 years, an average height of 175 cm, and a BMI averaging 28.7, categorizing them as overweight with obesity. Participants were monitored over 12 months wearing MADs. Prior to commencing therapy, all patients underwent polysomnographic evaluation at a sleep laboratory, yielding an AHI with a mean of 19.6 events per hour, ranging from a minimum of 5 to a maximum of 37 events per hour. Due to ethical considerations, cephalometric radiographs were not obtained from the participants.

The retrospective group comprised eight participants meeting the inclusion criteria of consistent MAD usage, availability of lateral cephalogram prior to MAD insertion, consent for new lateral cephalogram, and MAD wear for a minimum of two years from an initial pool of 16 patients, accounting for 31.2% unavailable or relocated and 18.8% declining additional X-rays. This group consisted of one female and seven male participants, averaging 59.1 years in age, 181.9 cm in height, and 27.2 in BMI, classified as overweight. The participants exhibited an average Apnea–Hypopnea Index (AHI) of 34.6 events per hour pre-therapy, ranging from 17 to 68 events per hour. The average duration of MAD usage at the time of screening examination was 7 ± 5.1 years. A repeat lateral cephalogram was deemed necessary to evaluate therapy-induced dentoskeletal changes.

The therapeutic protocol encompassed mandibular advancement via MAD by 50% of maximal protrusion, typically positioning patients in a head bite or exhibiting a narrow negative overjet, coupled with vertical locking of 6–8 mm.

### 2.2. Methodology of the Prospective Investigation

Patients in the prospective group underwent assessments during the first year post-MAD insertion, with follow-up visits scheduled at three-, six-, nine-, and twelve-month intervals. Initial evaluations included both extraoral and intraoral examinations along with impressions of both jaws. Additionally, impressions were retaken at each subsequent examination appointment. Model evaluations were conducted twice per examination date to enhance reliability, with the analyzed parameters being the SNA, SNB, ANB, NL-NSL, ML-NSL, ML-NL, IOK-NL, and IUK-ML angles as well as overjet and overbite.

**Figure 1:**
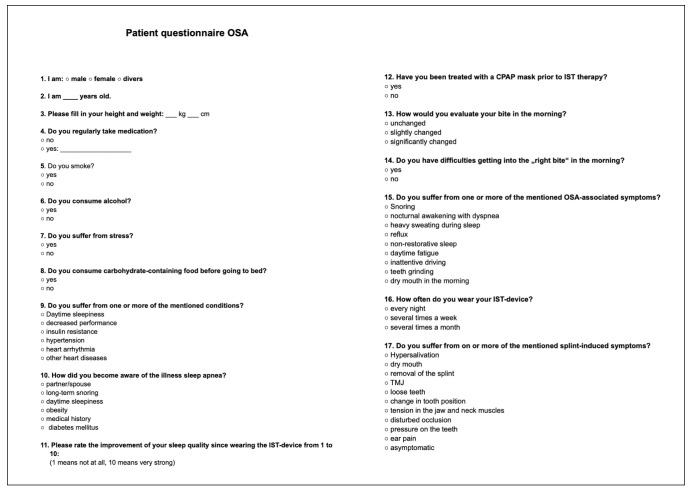
Patient questionnaire on OSA symptom- and MAD wearing-related aspects. Questionnaire comprising 14 inquiries covering general anamnestic information, as well as aspects associated with obstructive sleep apnea (OSA) and mandibular advancement device (MAD) usage.

At the three-month interval examination, participants completed a patient-specific questionnaire designed in accordance with the S3 guideline ‘Non-restorative Sleep/Sleep Disorders Chapter: Sleep-Related Breathing Disorders in Adults’, as well as the S1 guideline ‘The Mandibular Advancement Splint: Use in Dental Sleep Medicine in Adults’, which is shown in Figure 1. This dual-guideline framework both ensured the questionnaire’s alignment with established clinical standards and allowed for a thorough assessment of the participants’ experiences and outcomes related to their mandibular advancement device therapy.

The screening time points were T0—Before MAD therapy, T1—Screening appointment 1 (after 3 months), T2—Screening appointment 2 (after 6 months), T3—Screening appointment 3 (after 9 months), and T4—Screening appointment 4 (after 12 months).

### 2.3. Methodology of the Retrospective Investigation

Participants in the retrospective group underwent a single appointment (T1), during which they completed the patient-specific questionnaire and underwent lateral cephalogram image preparation. The measurement parameters for analyzing the radiographs included overjet and overbite, as well as angle class, and were calculated using the Z1 WinCeph and CellmatiQ programs.

The initial lateral cephalograms obtained before treatment begin (T0), which were already accessible, were compared with the newly generated X-ray images to assess changes over time.

### 2.4. Statistical Analyses

Statistical analysis was performed using Python 3.9.5 with libraries Pandas and NumPy for data exploration and processing, Matplotlib and Seaborn for plotting, and SciPy. The paired *t*-test was applied to evaluate the significance of the mean difference between paired observations in the retrospective group, as was ANOVA. The significance level was set at *p* < 0.05.

## 3. Results

### 3.1. Questionnaire

The questionnaire analysis revealed a high tolerance for MADs, with nearly 70% of the prospective group and slightly over 70% of the retrospective group wearing them nightly, while the remaining participants in both cohorts used the devices several times a week, without any reporting less frequent usage. The survey results indicated that approximately 40% of the prospective group and 60% of the retrospective group observed a slight morning alteration in occlusion, with only less than 10% of the prospective group reporting a significant change, while the rest noted no change. Regarding MAD-induced symptoms, most common issues included hypersalivation, tension in jaw and neck muscles, pressure on teeth, dry mouth, and temporomandibular joint dysfunction (TMD), with the prospective group mentioning the first three more frequently.

Furthermore, unanimous consensus among participants from both the prospective and retrospective cohorts indicated a notable amelioration in sleep quality consequent to MAD therapy on a scale of 1 = no improvement to 10 = extreme improvement, with a predominant proportion in the prospective group endorsing a rating of 9, closely followed by 7, while in the retrospective group, over 10% rated it a perfect score of 10 on the aforementioned scale delineating improvement.

**Figure 2:**
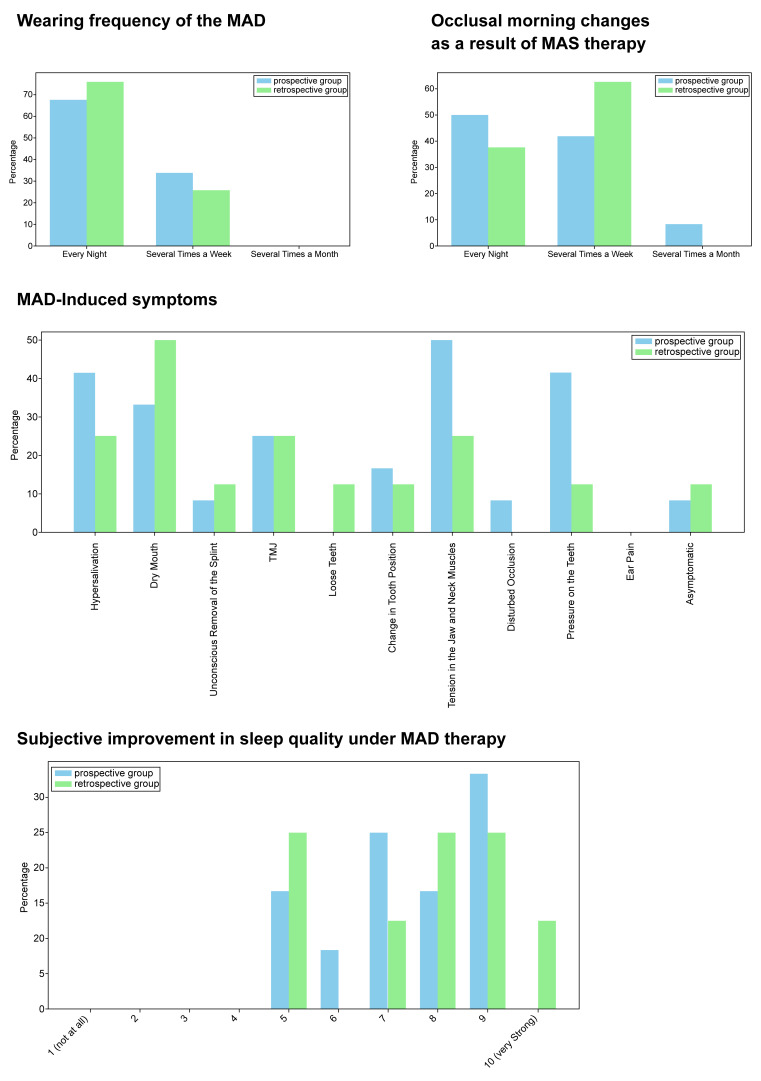
Most important outcomes of the questionnaire. Principal findings from analyzing patient responses to the questionnaire encompassing the frequency of MAD utilization, evaluation of occlusal morning alterations resultant from MAD therapy, identification of MAD-induced symptoms, and subjective amelioration in sleep quality during MAD intervention.

The key findings of the questionnaire evaluation are depicted in Figure 2.

### 3.2. Analysis of the Models in the Prospective Group

The statistical analysis demonstrated significant alterations in both the overjet and overbite measurements obtained from the models over the course of the study. Regarding overjet, the hypothesis that there would occur notable changes in this parameter at each individual screening session compared to the preceding one could be mostly confirmed. At T1, there was no significant difference in overjet compared to the baseline measurement at T0 prior to the commencement of therapy. However, after six months (T2), a highly significant change was noted compared to the measurement taken three months prior with a reduction in overjet (*p* = 0.00066). This trend persisted with another significant reduction observed at nine months (T3) compared to the previous measurement (*p* = 0.00337). Subsequently, at twelve months (T4), a further diminishment in overjet was identified compared to the measurement at nine months (*p* = 0.01947). Initial overjet measurements averaged 2.9 ± 1.4 mm pre-treatment, declining to 2.1 ± 1.1 mm at the conclusion of assessments at T4.

Concerning overbite, it was likewise hypothesized that the use of the MAD would induce noteworthy alterations in this parameter at each appointment. The results of the analyses indicated a significant change in overbite between T0, directly preceding the initiation of therapy, and T1, following 3 months of device wear, in terms of a deepening of the bite, with a *p*-value of 0.0261. This trend persisted at T2 after 6 months of device usage where the overbite again exhibited a significant change compared to the measurement taken 3 months earlier (*p* = 0.0203). In contrast to the overjet, no further significant alterations were observed for the overbite after 9 (T3) and 12 months (T4). Thus, the change noted at T2 persisted. The initial pre-treatment overbite measurements were recorded at 2.3 ± 1.7 mm, increasing slightly to 2.5 ± 0.3 mm at the conclusion of the examinations (T4).

**Figure 3:**
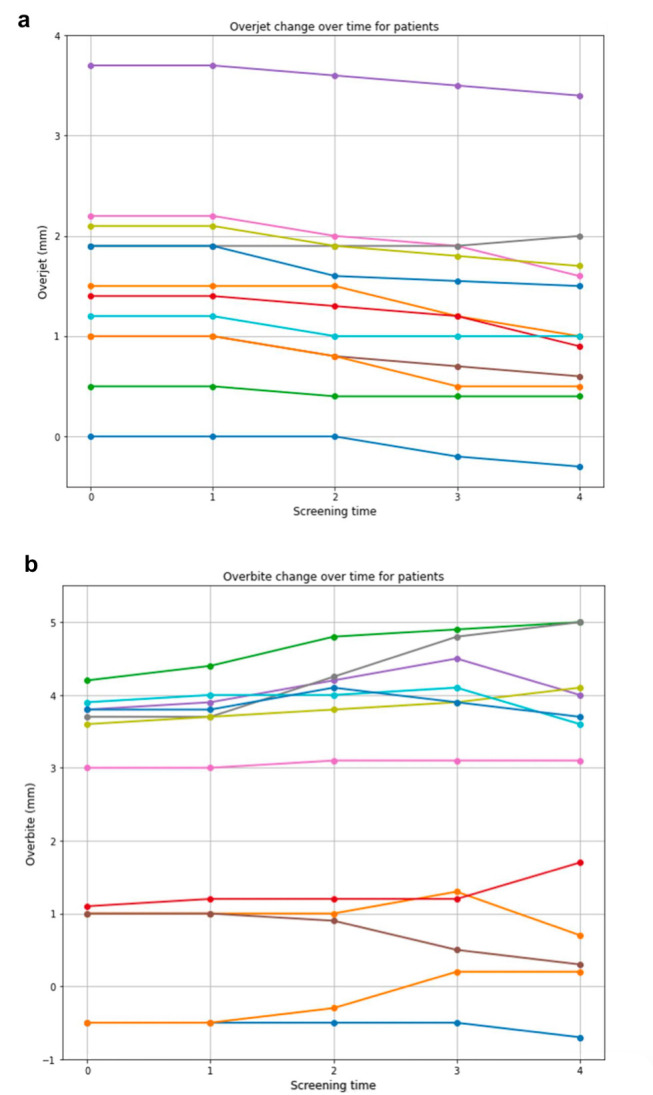
Overjet and overbite changes of the prospective group investigated in the models. Overjet (**a**) and overbite (**b**) changes in mm were investigated in the models for each screening time point. T0—Initial visit before UPS therapy, T1—Screening appointment 1 (after 3 months), T2—Screening appointment 2 (after 6 months), T3—Screening appointment 3 (after 9 months), and T4—Screening appointment 4 (after 12 months). Each color line corresponds to an individual patient.

Graphical representations of overjet and overbite changes in mm for each patient across all screening periods are provided in Figure 3.

Additionally, the models underwent further analysis to assess changes in angle class in the left and right canine and molar regions. However, no significant alterations were observed at any of the screening time points.

### 3.3. Analysis of the Lateral Cephalograms in the Retrospective Group

The analysis of lateral cephalograms in the retrospective group provided crucial insights into the effects of long-term treatment with MADs. Regarding anticipated changes in overjet between the initial record taken before start of the treatment and the screening after at least two years of device wearing and a mean value of 7 years for the group, a significant reduction in overjet between the baseline screening and the follow-up assessment could be noted, underscoring the results obtained from the prospective patient group (*p* = 0.00409) and substantiating the therapeutic impact of MAD therapy over an extended duration. Overjet measurements mirrored those of the prospective group pre-MAD insertion, registering at 2.9 ± 2.1 mm, subsequently decreasing by over fifty percent to 1.4 ± 1.1 mm over the course of long-term treatment.

**Figure 4:**
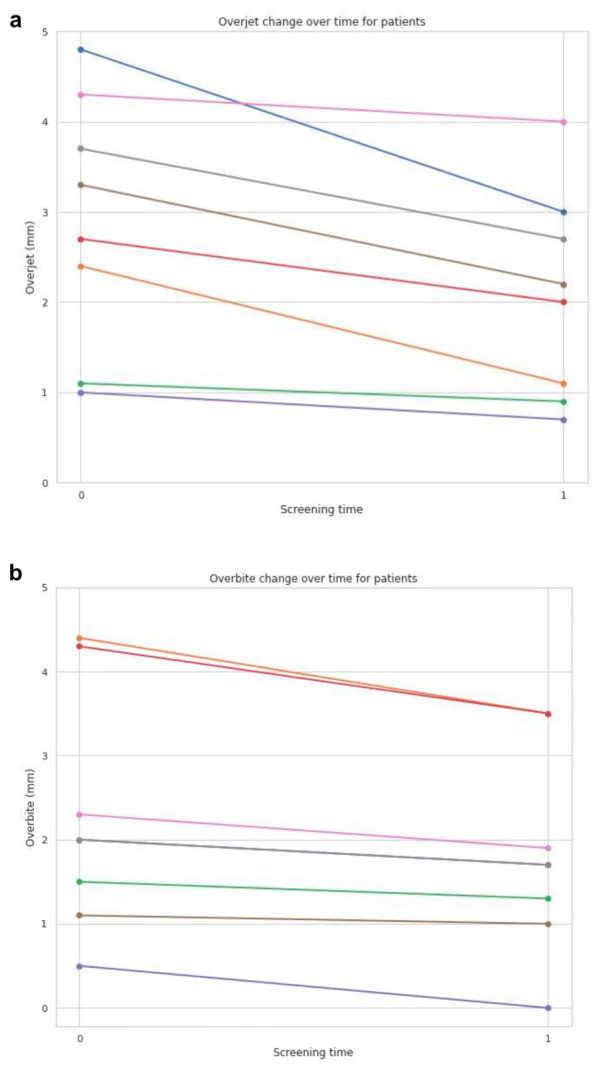
Overjet and overbite changes of the retrospective group investigated on the lateral cephalograms. Overjet (**a**) and overbite (**b**) changes in mm were investigated on the lateral cephalograms for each screening time point. T0—Initial visit before UPS therapy and T1—Screening appointment after at least 2 years of therapy. Each color line corresponds to an individual patient.

Similarly, the hypothesis of observing discernible changes in overbite between the initial and the subsequent screening could be observed. At screening T1, the derived *p*-value of 0.00323 relative to T0 elucidated a notable shift in overbite, however, contrarily to the results of the prospective group effecting an overbite reduction over the prescribed treatment duration. The overbite measurements were recorded as 2.3 ± 1.4 mm at time T0, decreasing to 1.8 ± 1.2 mm at T1. Figure 4 graphically illustrates the changes in overjet and overbite measured in millimeters for each patient across screening periods.

**Figure 5:**
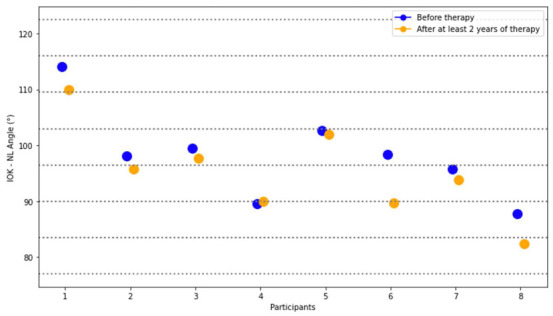
Change in IOK-NL angle of the retrospective group investigated on the lateral cephalograms. Change in IOK-NL angle before treatment (blue dots) and after long-term therapy with MADs (yellow dots) for each patient participating in the retrospective study group in degree (°).

The assessment of lateral cephalograms regarding changes in measured angles again provided insights into the impact of MADs as treatment interventions over a prolonged duration. At this, it was revealed that only the IOK-NL Angle evaluating the axial position of the maxillary tooth relative to the maxilla exhibited statistically significant variances between pre-therapy with a mean value of 98.2° ± 8.1° and 95.2° ± 8.4° at follow-up measurements (*p* = 0.02017). This alteration precipitated a decrease in the angle, manifesting as a retroclination of the upper anterior teeth. Contrarily, the remaining angles assessed in the lateral cephalograms displayed no significant deviations over the treatment period, emphasizing the specificity of treatment effects on the IOK-NL angle in this patient cohort. Changes in IOK-NL angle for each patient investigated are presented in Figure 5.

## 4. Discussion

MADs have emerged as valid treatment devices favored in mild to moderate OSA and in cases of CPAP intolerance due to improved patient compliance and tolerability. Nonetheless, MAD treatment can yield significant occlusal side effects, such as the retroclination of the upper incisors, proclination of lower incisors, and alterations in overjet and overbite, potentially leading to therapy discontinuation [27]. Although these effects are already known and described in the literature, there is still a lack of data elucidating the precise time point of occurrence, the progression in severity, and the long-term development of these side effects, further taking into account their severity and the correlation with patient compliance and long-term therapy continuation.

To bridge this gap, this study investigated the efficacy and orthodontic effects of MADs utilizing both prospective and retrospective study designs to comprehensively assess treatment outcomes, focusing on usage patterns, symptomatic outcomes, and occlusal changes over various time intervals. While in the prospective group assessments conducted at regular short-term intervals of three months served to identify earliest onset and progression mode of occurring side-effects, the retrospective group consisting of patients with long-term MAD usage allowed for an evaluation of sustained treatment effects. The questionnaire analysis indicated a high tolerance for MADs in both groups, with the majority of participants wearing them nightly, leading to notable improvements in sleep quality. However, common MAD-induced symptoms included hypersalivation and TMJ dysfunction, though these were generally manageable. These findings resonate with complementary research on the enduring repercussions of MAD therapy, delineating the induction of alterations in occlusion, orofacial functionality, and TMJ dynamics. However, concurrently, they underscore the manageable nature of these sequelae, given the pronounced therapeutic advantages conferred by MAD therapy [28]. The high long-term acceptance of wearing the devices finds reinforcement by additional investigations that have found high long-term adherence in patients still wearing their devices one month following therapy commencement, which underscores the rapid adaptability of individuals to MADs [29].

This investigation additionally revealed that MAD therapy elicits notable changes in overjet and overbite; however, due to the small sample size, these results should be interpreted with caution, as they may not fully represent the broader population or capture the variability that could be present in a larger cohort. Overjet exhibited a pronounced decrease after six months of therapy among the prospective group, with a continuing reduction throughout the twelve-month screening period. This was corroborated by findings from the retrospective group, indicating a sustained decrease in overjet as a long-term effect. Examination of lateral cephalograms revealed a reduction in the IOK-NL angle, indicating retroclination of the upper anterior teeth, likely accounting for this effect, as there were no alterations noted in angle class based on model analysis. In contrast to the consistent findings regarding overjet changes across both groups, alterations in overbite showed contrasting patterns. In the prospective group, an initial deepening of the bite was observed after three months of device usage, which stabilized thereafter. Conversely, a significant reduction in overbite was observed in the retrospective group as a long-term effect. This initial effect of bite deepening may be attributed to a phenomenon akin to the impact of aligners on occlusion [30]. During treatment with aligners, intrusion movements are commonly observed over the treatment period, whereat this phenomenon, often termed the “bite block effect”, is attributed to the combined influence of aligner thickness and occlusal forces. However, these study findings further demonstrate that the intrusive effects diminish with prolonged use, while conversely, an observed reduction in overbite attributed to the biomechanical long-term impact of MADs occurs. These findings, along with a reduction in overjet and a retroclination of the upper incisors align with prior research examining the impact of MADs on dental alignment [27,31,32]. Contrary to our results, however, these studies also describe a significant protrusion of the lower incisors. This variation observed in comparison to other studies could be attributed to the varying dimensions of the mandibular protrusion within devices, with recommendations varying significantly in terms of maximum protrusion capacity, from 6% to 90% [33]. In this study, 50% of maximum protrusion was employed as standard, adhering to the recommendation that the therapeutic MAD position should prioritize patient comfort by minimizing pain or tension, ideally converging with approximately 50% of the maximum protrusion as outlined in the Diagnostic Criteria for Temporomandibular Disorders (DC/TMD), amidst concurrent investigations applying the same protrusion extent [26,34,35]. However, in certain research endeavors, the devices were only initially installed with 50% of the maximum mandibular protrusion capacity, progressively advancing until reaching the therapeutic protrusion, which varied between 85 to 100% of the maximum mandibular protrusion [34,36]. In this study, 50% of the maximum protrusion was deliberately chosen and maintained for the entire treatment period, based on the previously mentioned recommendation of the DC/TMD and considering the observed correlation between protrusion extent and maxillary anterior tooth inclination, as noted in prior research [32]. At this, the absence of significant proclination of lower incisors observed in both the prospective and retrospective study groups might also be attributed to this moderate advancement, which, despite its modest nature, proved to be adequate in effectively treating OSA. This hypothesis is supported by a long-term study which also applied a constant protrusion of 50% of the maximum capacity and recorded only clinically insignificant dentoskeletal changes under MAD therapy [37]. However, it is important to reiterate again that, given the small sample size in this investigation, these results should be interpreted with caution as the limited numbers may restrict the generalizability of these findings and could obscure potential variability that might be evident in a larger population.

This study elucidates several limitations and implications for clinical practice that merit rigorous examination. Firstly, the generalizability of these findings is constrained by the small sample size, which is not representative of the broader population. Future research should aim to include a diverse demographic to enhance the applicability of the results across a wider array of patients. Secondly, the reliance on patient-reported outcomes concerning tolerability and side effects may introduce bias, as patients might underreport symptoms influenced by various factors, including the perceived effectiveness of the treatment. Incorporating objective measures and clinician assessments could provide a more comprehensive evaluation of treatment outcomes. Furthermore, while the study focuses on specific outcomes and parameters such as overjet and overbite, it did not address the full range of implications due to MAD therapy crucial for assessing the full impact of treatment.

Although blinding was not implemented during the measurement process, all data were collected by a single investigator and the small variation in measurements at the millimeter or submillimeter level may reduce the possibility of significant bias.

From a statistical perspective, ANOVA (Analysis of Variance) could have been a robust alternative to the t-test applied in this study when comparing means across multiple groups, as it allows for the simultaneous assessment of differences between more than two groups while controlling for type I error inflation. However, it was not used in this study due to its need for a large sample size and assumptions of homogeneity and normality, which were not met here. Instead, the t-test was selected as a more appropriate statistical approach, ensuring greater reliability and validity in the context of this study’s design and data constraints.

It needs to be emphasized that the comparison of retrospective and prospective outcomes warrants careful scrutiny due to inherent biases in data collection methodologies. Furthermore, the limited sample sizes in both cohorts significantly attenuate statistical power, heightening the risk of Type I and Type II errors and undermining the generalizability of the results. Consequently, the observed trends should be interpreted with caution, necessitating further investigation with larger and more rigorously designed studies to validate these findings.

The implications for clinical practice derived from this study suggest the necessity of individualized treatment protocols. The findings indicate that maintaining a moderate degree of mandibular protrusion at 50% of the maximum may mitigate certain adverse effects while still delivering effective OSA treatment. Clinicians are encouraged to tailor protrusion levels to individual patients based on their unique needs and tolerability. Additionally, regular monitoring of dental and occlusal changes during MAD therapy is imperative. Clinicians should schedule follow-up appointments to evaluate any emerging side effects and intervene as necessary to prevent treatment discontinuation. By adopting a more comprehensive and individualized approach, clinicians can enhance patient outcomes and ensure the viability of MAD therapy for individuals suffering from OSA. This study also highlights the need for future research to explore alterations in MAD design that could alleviate pressure on anterior teeth and minimize occlusal misalignments. This may involve designing appliances that exclude anterior components, thus reducing potential side effects while maintaining therapeutic efficacy. Considering the complex interplay between dental health and sleep apnea treatment, a multidisciplinary approach involving dentists, orthodontists, and sleep specialists may optimize patient outcomes, as collaborative care can enhance patient adherence and improve overall management strategies for OSA. Lastly, there is a clear need for further studies to evaluate the long-term effects of MAD therapy on dental health, patient compliance, and sleep quality in order to inform clinical guidelines and help refine treatment protocols.

In summary, this study diverges from prior research positing that MADs harbor significant side effects, as the results unveiled here exhibited a paucity of adverse effects. MADs proved to be potent therapeutic tools, notably enhancing sleep quality and securing high patient adherence rates, thus ensuring commendable compliance due to the prevailing perceived benefits. Despite the presence of occlusal and general side effects, patients exhibited complete tolerance towards them on the grounds of these substantial advantages. While statistical analysis indicates minor dental repercussions such as alterations in overjet, overbite, and maxillary anterior tooth inclination, their clinical insignificance renders them negligible. Notably, the absence of changes in angle class or mandibular anterior tooth protrusion, unlike prevalent findings in extant literature, may be attributed to the deliberate maintenance of moderate therapeutic mandibular protrusion throughout the entire treatment. Despite its modest degree, this moderate advancement proved sufficient in effectively treating OSA, laying the groundwork for a recommendation endorsing a moderate mandibular protrusion strategy. However, it is essential to approach these findings with caution, as the small sample size may limit their generalizability and may not capture the full spectrum of patient responses. Furthermore, considering that changes mainly occur in the front teeth, it is imperative for forthcoming studies to prioritize exploring adjustments in appliance design. This could involve omitting frontal structures from the appliances to reduce pressure on these areas, thereby preventing potential occlusal misalignments.

## 5. Conclusions

In conclusion, this study illuminates the effectiveness of MADs in treating OSA, showing minimal adverse effects despite anticipated occlusal changes and dental alignment alterations. Investigations exhibited MADs as potent therapeutic tools, significantly improving sleep quality and securing high patient adherence rates, thereby ensuring very high compliance. While minor dental repercussions were noted, their clinical insignificance renders them negligible in the context of the substantial therapeutic benefits conferred by MAD therapy. This work diverges from prior research by maintaining a moderate therapeutic mandibular protrusion throughout treatment, which proved effective in addressing OSA while minimizing adverse dental effects. Notably, the absence of changes in angle class or mandibular anterior tooth protrusion underscores the importance of this moderate advancement strategy. Moving forward, future research should prioritize investigating appliance design modifications aimed at mitigating pressure exerted on anterior teeth, thus preventing subsequent occlusal deviations. Overall, these findings support the continued use of MAD therapy as a valuable treatment option for OSA, emphasizing the importance of balancing therapeutic efficacy with dental health considerations.

## Data Availability

The raw data will be made available by the authors on request.
